# OCTA in age-related macular degeneration: consensus on practical guidelines for optimal imaging strategies across different clinical scenarios

**DOI:** 10.1007/s00417-026-07194-4

**Published:** 2026-03-14

**Authors:** Giovanni Neri, Daniela Bacherini, Rodolfo Mastropasqua, Rosa Dolz-Marco, Roberto Gallego-Pinazo, Gregor S. Reiter, Michele Reibaldi, Enrico Borrelli

**Affiliations:** 1 Department of Surgical Sciences, University of Turin, Turin, Italy; 2 Department of Ophthalmology, “City of Health and Science” Hospital, Turin, Italy; 3 Department of Neurosciences, Psychology, Drug Research, and Child Health, Eye Clinic, University of Florence, Azienda Ospedaliero Universitaria Careggi, Florence, Italy; 4 Department of Neurosciences, Imaging and Clinical Sciences, University “G. d’Annunzio” Chieti-Pescara, Chieti, Italy; 5 Unit of Macula, Oftalvist Clinic, Valencia, 46004 Spain; 6 Department of Ophthalmology and Optometry, Medical University of Vienna, Vienna, Austria; 7 Department of Ophthalmology, Mayo Clinic, Rochester, MN USA; 8 Division of Ophthalmology, Department of Surgical Sciences, University of Turin, Via Cherasco, 23, Turin, Italy

**Keywords:** Age-related macular degeneration, Optical coherence tomography angiography, Imaging, Macular neovascularization, Geographic atrophy

## Abstract

**Purpose:**

To provide expert-driven, practical guidelines for the optimal use of optical coherence tomography angiography (OCTA) in the clinical management of age-related macular degeneration (AMD). Given the heterogeneity of AMD and the versatility of OCTA, clinicians require scenario-specific recommendations to navigate imaging strategies effectively across various stages and phenotypes of the disease.

**Methods:**

An expert panel from the European Young Retinal Imaging and Retina Study Group conducted a consensus-based review integrating the latest evidence and extensive clinical experience. The panel focused on developing OCTA-based protocols tailored to distinct clinical scenarios in AMD, including neovascular subtypes, geographic atrophy, non-exudative neovascularization, and ambiguous fluid accumulations. Recommendations emphasize appropriate scan sizes, slab selections, and strategies for interpreting flow signals across disease presentations.

**Results:**

The expert panel delineated structured OCTA protocols for a range of AMD phenotypes to enhance diagnostic accuracy and clinical decision-making. In neovascular AMD, OCTA enables detailed characterization of macular neovascularization (MNV) subtypes—types 1, 2, and 3—through high-resolution, depth-resolved imaging. Recommendations include the use of cross-sectional OCTA to localize flow and appropriate selection of *en face* slabs to optimize lesion visualization. In geographic atrophy (GA), while structural OCT remains the primary modality, OCTA may assist in identifying subclinical or complicating MNV and in assessing choriocapillaris perfusion in the peri-atrophic zone, which may have prognostic implications. In cases of early and intermediate AMD, OCTA plays a critical role in detecting non-exudative MNV—particularly type 1 and type 3 lesions—providing valuable prognostic information given the risk of exudative conversion. Additionally, OCTA contributes to the differential diagnosis of pseudovitelliform lesions, detection of deep retinal age-related microvascular anomalies (DRAMAs), and exclusion of neovascularization in cases presenting with atypical intraretinal or subretinal fluid.

**Conclusions:**

This expert consensus offers a pragmatic framework for applying OCTA in AMD clinics, advocating for individualized imaging approaches based on lesion type and anatomical complexity. By enhancing diagnostic precision and enabling tailored monitoring strategies, OCTA integration into clinical workflows may significantly improve patient outcomes across the AMD spectrum.

## Key Messages

*****What is known***:**

- OCTA has significantly enhanced the clinical management of patients with age-related macular degeneration (AMD), a heterogeneous condition characterized by various challenges and lesion types

*****What is new***:**

- This study presents an expert consensus on the practical use of OCTA in the clinical management of AMD
- While OCTA is a powerful and effective tool in this context, optimal use requires awareness of specific considerations and limitations

## Introduction

Age-related macular degeneration (AMD) is a leading cause of vision impairment and legal blindness globally, and the primary cause in individuals over 55 in Western countries [1]. Vision loss is most commonly associated with the late stages of AMD, which include neovascular AMD and geographic atrophy (GA) [2].

For many years, fluorescein angiography (FA) and indocyanine green angiography (ICGA) have been regarded as the gold standards for diagnosing and monitoring AMD, particularly for confirming or excluding the presence of macular neovascularization (MNV), the hallmark feature of neovascular AMD [3, 4]. FA is based on the injection of fluorescein dye, which binds to plasma proteins with approximately 80% affinity and emits fluorescence at wavelengths between 520 and 530 nm [5]. Conversely, ICGA is based on the injection of indocyanine green dye, which has a higher protein-binding affinity of about 95% and fluoresces in the near-infrared spectrum, allowing image acquisition at around 835 nm [6]. Thanks to its longer wavelength, ICGA provides enhanced visualization of the choroidal vasculature, as the retinal pigment epithelium (RPE) absorbs the shorter wavelengths used in FA. Additionally, the lower protein-binding capacity of fluorescein results in more leakage from both normal and abnormal vessels compared to indocyanine green. Consequently, FA is primarily suited for evaluating the retinal circulation, whereas ICGA is more effective for assessing the choroidal circulation.

Although still widely used and well established, these techniques have several limitations: they are invasive, requiring intravenous dye injection, which can lead to side effects such as nausea and vomiting, and in rare cases, severe allergic reactions and are time-intensive, often occupying equipment in busy clinical practice. Furthermore, they do not provide detailed visualization of the deeper vascular layers [7–9]. FA and ICGA are typically used in conjunction with structural optical coherence tomography (OCT), an imaging modality that provides volumetric structural information of the retina, retinal pigment epithelium (RPE), and choroid. This allows for the rapid, non-invasive acquisition of key data essential for the diagnosis and monitoring of patients with all stages of AMD [10].

In recent years, the understanding of AMD has significantly advanced with the introduction of optical coherence tomography angiography (OCTA) [11–14]. OCTA enables non-invasive visualization and analysis of the retinal and choroidal vasculature.[12, 13, 15, 16] This technique acquires repeated B-scans -composed of multiple A-scans at the same retinal location- to detect changes in signal intensity, known as decorrelation signals [17]. Since the retina is a static tissue, these decorrelation signals are attributed to blood flow within the retinal and choroidal vessels [18].

Most commercially available OCTA systems are based on either spectral-domain (SD) or swept-source (SS) technology. Compared to SD-OCTA, SS-OCTA devices utilize longer wavelengths, around 840 and 1050 nm respectively, which allow for deeper light penetration through the RPE, resulting in improved visualization of the choroid [19, 20]. Moreover, OCTA scan areas typically range from 2 × 2 mm to 12 × 12 mm, with 3 × 3 mm and 6 × 6 mm scans, generally consisting of 304 and 512 B scans respectively, being the most commonly used in clinical practice, as image quality generally declines with increasing scan size [21].

OCTA provides both cross-sectional and en face images. In cross-sectional OCTA B-scans, flow signal information is superimposed on the structural data. In contrast, *en face* OCTA images display only flow signal information, without the underlying structural context. Whether using cross-sectional or *en face* images, OCTA—unlike FA and ICGA—enables depth-resolved analysis of the retinal and choroidal vasculature [18]. To generate *en face* OCTA images, two segmentation boundaries typically defined a slab, and the flow information between them is visualized creating a reconstruction of the vessels located within the margins of the slab. Most OCTA devices offer automated segmentation, with the option to manually adjust these boundaries when needed [22]. Automatic segmentation is typically used in both clinical studies and practice due to the significant time required for manual segmentation, which is generally reserved for specific cases [22].

OCTA devices commonly provide automatically generated slabs for layer-specific analysis of the retinal and choroidal vasculature:

- The superficial retinal slab extends from the inner limiting membrane (ILM) and includes the ganglion cell layer (GCL) and inner plexiform layer (IPL). It visualizes the superficial capillary plexus and typically includes part of the radial peripapillary capillary plexus.
- The deep retinal slab extends from the IPL to the outer plexiform layer (OPL), encompassing the intermediate and deep capillary plexuses (i.e., deep vascular complex - DVC, when combined), which may also be visualized separately with some devices.
- The avascular outer retinal slab covers the OPL and the outer nuclear layer (ONL). As the name suggests, this slab is avascular in healthy individuals.
- The choriocapillaris slab typically consists of a layer located just below Bruch’s membrane. The thickness of this layer may vary depending on the device employed.
- Several OCTA devices enable separate evaluation of the radial peripapillary capillary (RPC) network, which is the most superficial vascular plexus. This network is located within the retinal nerve fiber layer (RNFL), primarily in the peripapillary region [23].
- Given their clinical relevance, many devices also provide additional predefined slabs. These include the outer retina to choriocapillaris (ORCC) slab, which combines the avascular outer retina and choriocapillaris layers, and the RPE–RPEfit slab, which spans from the RPE to the bottom of Bruch’s membrane [18].

Therefore, OCTA offers a variety of scan types and visualization modes, providing a versatile tool for assessing vascular lesions in AMD. This flexibility is a significant advantage, as it enables a comprehensive assessment. However, it also presents a challenge as clinicians must decide which imaging strategy is most appropriate for each specific clinical scenario in patients with AMD.

The aim of this review is to serve as a practical guide for optimizing the use of OCTA in routine clinical practice for patients with AMD. We outline several common clinical situations encountered in AMD management and provide recommendations for the most suitable strategies in each case. To do so, two authors (GN and EB) met to define the clinical scenarios and the most appropriate methodological approach for each. The resulting practical guidelines were then shared with the remaining authors and revised based on their feedback until consensus was reached for each scenario. It is important to recognize that different devices may employ different segmentation strategies, and that manual modification of segmentation boundaries is not always possible. This may lead to heterogeneity between devices, potentially affecting the applicability of the proposed guidelines.

## Exudative neovascular AMD

Exudative neovascular AMD is marked by the growth and leakage of MNV. MNV can be classified into three main types: type 1, type 2, and type 3 [24]. Additionally, aneurysmal type 1 (AT1) MNV—also referred to as polypoidal choroidal vasculopathy (PCV)—is considered a subtype of type 1 MNV. While less frequently observed in AMD, this form is more commonly associated with pachychoroid disease, particularly in Caucasian populations [25, 26].

Type 1 MNV is characterized by the proliferation of neovessels originating from the choroid and extending into the sub-RPE space, accompanied by fibroblasts, myofibroblasts, and macrophages, which may contribute to fibrotic tissue formation.[27] It is the most prevalent form of MNV in AMD, representing approximately 40% of cases [28].

Historically, type 1 MNV was primarily diagnosed using FA and ICGA. On FA, type 1 MNV typically appears as an area of punctate hyperfluorescence, with poor delineation of the neovascular network—hence the term “occult choroidal neovascularization (CNV)”[27]. In this context, ICGA offers improved visualization, as type 1 MNV generally appears as a hyperfluorescent area in the late phases of the examination.

Currently, type 1 MNV is typically suspected based on structural OCT findings, where the lesion is located beneath the RPE layer with variable associated signs. It may appear as a shallow and irregular pigment epithelial detachment (PED) with internal mixed reflectivity[29]—commonly referred to as the double layer sign (DLS) or as the shallow, irregular RPE elevation (SIRE) [30] — composed by Bruch’s membrane externally and RPE internally, or present as a higher PED with variable serous and fibrovascular components. In the presence of type 1 MNV, the DLS is typically more pronounced (i.e., thick DLS), with increased separation between Bruch’s membrane and the RPE compared to cases with similar OCT features but without MNV [31]. When exudation is present, these PEDs are usually accompanied by OCT features of exudation, such as intraretinal fluid (IRF), subretinal fluid (SRF), bacillary detachment or subretinal hyperreflective material (SHRM) [32].

**Fig. 1:**
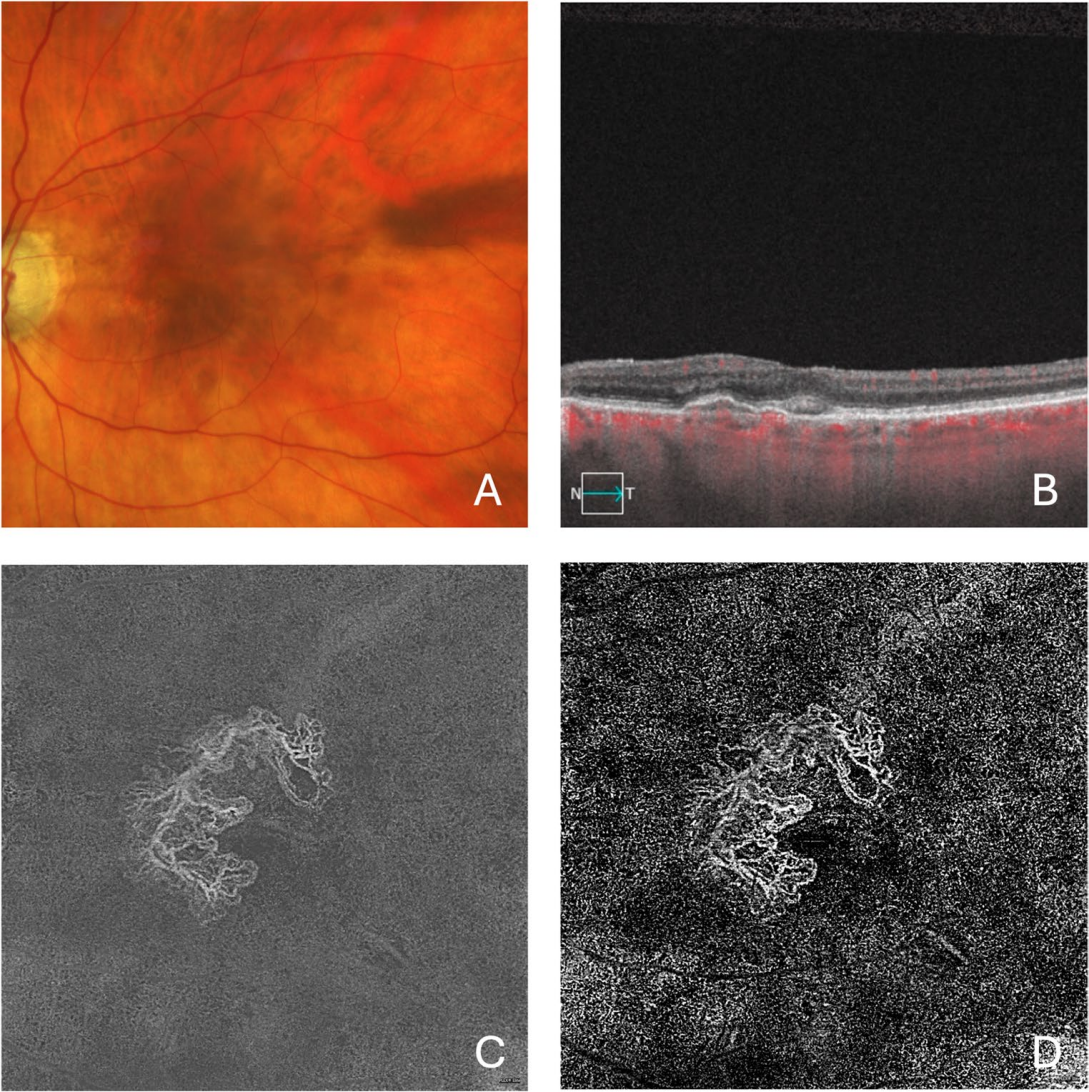
Multimodal imaging of the left eye from a patient with neovascular AMD and type 1 MNV. The color fundus image (**A**) displays macular pigment abnormalities and irregularities. The OCTA B-scan (**B**) provides structural detail in grayscale, with flow signals overlaid in red; it reveals a pigment epithelium detachment accompanied by subretinal fluid. The OCTA ORCC (C) and RPE–RPE fit (D) slab en face images highlight an extensive neovascular network

When a shallow PED is present, OCTA is essential to confirm the presence of type 1 MNV, as other lesions, such as basal laminar deposits (BLamD), may mimic the same OCT features, appearing as a shallow, irregular PED with thin DLS [33, 34]. To verify the presence of type 1 MNV in such cases, a 3 × 3 mm or 6 × 6 mm OCTA scan is recommended, as both sufficiently cover the macular region and offer high detail resolution (Fig. 1). The initial step for detecting type 1 MNV is to assess cross-sectional OCTA images to identify flow signal between the RPE and Bruch’s membrane, overlapping with the hyperreflective material forming the thick DLS. It is important to note that some devices do not permit removal of projection artifacts during this visualization, which can affect the accuracy of the assessment. Subsequently, *en face* visualization provides the best overview of type 1 MNV. Two *en face* slabs can be used: the ORCC slab and the RPE–RPEfit slab. While the RPE–RPEfit slab enhances the contrast between MNV and surrounding tissue [35]—making MNV more conspicuous—the ORCC slab may be clinically advantageous, as it can also capture any type 2 MNV components located above the RPE, which would otherwise be missed using the RPE–RPEfit slab.

As mentioned above, while type 1 MNV often appears as a thick DLS/SIRE, it can also present as a taller PED with varying serous and fibrovascular components. In such cases, OCTA faces challenges, particularly when a tall PED has a large serous component. Segmentation algorithms may fail to accurately delineate the lesion, and importantly, the serous fluid can obscure the underlying MNV. When a serous PED is present, it is common for the type 1 MNV to be located at the lesion’s edge. A useful approach is to examine the shallow PED at the border and use OCTA to detect any flow signal within this area.

Aneurysmatical type 1 MNV is a subtype of type 1 MNV that commonly arises in the context of pachychoroid disease, although it can also occur in patients with AMD—more frequently among Asian populations. On structural OCT, AT1 MNV typically presents as a shallow, irregular PED with internal mixed reflectivity, corresponding to the type 1 neovascular component. This is accompanied by a dome-shaped PED at the margins, within which an oval-shaped hyperreflective lesion with a hyporeflective core can frequently be seen, representing the aneurysmal (polypoidal) structure. As with type 1 MNV, a 3 × 3 mm or 6 × 6 mm OCTA scan is recommended for evaluating aneurysmal type 1 MNV. Both B-scan and en face OCTA visualizations are useful in this context. On OCTA B-scans, flow can often be detected between the RPE and Bruch’s membrane, as well as within the aneurysmal (polypoidal) structures. En face views using ORCC or RPE–RPEfit slabs are helpful to visualize the neovascular network, often revealing a round hyperreflective lesion corresponding to the aneurysmal component. However, it is important to note that the aneurysmal lesion may not always display detectable flow on OCTA, likely due to the presence of high-flow turbulence, which can fall below the detection threshold of the technology. In this context, it is important to note that multiple aneurysmal lesions may occasionally be present, potentially affecting both prognosis and therapeutic approach—for instance, by warranting the addition of photodynamic therapy (PDT) to conventional anti-VEGF treatment.

Type 2 MNV involves the growth of new blood vessels originating from the choroid, similar to type 1 MNV. However, in type 2, these vessels emerge through the eroded RPE and predominantly proliferate within the subretinal space [27]. Among the MNV subtypes associated with AMD, pure type 2 is the least common, representing approximately 9% of cases, however type 2 lesions frequently associate with type 1 lesions in mixed 1/2 MNV [28]. On FA, type 2 MNV usually appears as a well-defined area of hyperfluorescence in the early phases, followed by dye leakage in the later stages “classic lesions” [27]. On structural OCT, the presence of SHRM should raise suspicion for type 2 MNV. However, SHRM is a nonspecific OCT finding that can also be associated with other lesions, such as vitelliform material, exudation or subretinal hemorrhage, in addition to neovascular membranes [36].

**Fig. 2:**
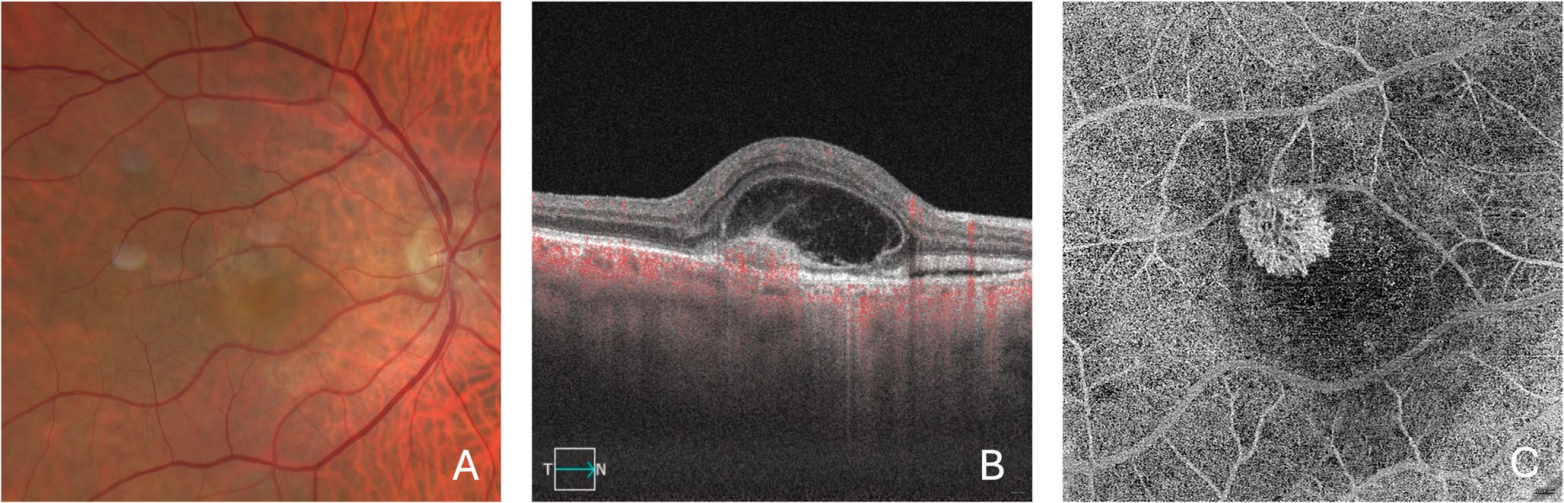
Multimodal imaging of the right eye from a patient with AMD and type 2 MNV. The color fundus photograph (**A**) reveals macular irregularities with pigmentary changes. The OCTA B-scan (**B**) confirms subretinal hyperreflective material (SHRM) containing flow, along with associated intraretinal and subretinal fluid. The OCTA ORCC slab en face image (**C**) clearly displays a neovascular network

OCTA can be a valuable tool to determine whether SHRM contains neovascular tissue, thereby confirming the diagnosis of type 2 MNV (Fig. 2). A 3 × 3 mm or 6 × 6 mm OCTA scan is recommended, as both adequately cover the macular area while providing high-resolution detail. The evaluation should begin with cross-sectional OCTA images to detect flow signals within the SHRM. Once flow is confirmed, *en face* visualization offers the best overview of type 2 MNV. The optimal slab for this purpose is the ORCC slab, as it fully encompasses the SHRM—typically located in the outer retina—and may also reveal any component of the MNV that extends beneath the RPE.

Type 3 MNV is the second most common form of MNV in AMD, accounting for approximately 34% of cases [28]. Type 3 MNV was first identified by Hartnett et al. [37] in 1992 and termed “retinal vascular abnormality”, and was later referred to as “retinal angiomatous proliferation” (RAP) by Yannuzzi et al. [38]. This subtype of MNV is characterized by the downward proliferation of vessels originating from the DVC toward the outer retina [27, 39–42]. On structural OCT, type 3 MNV typically appears as a hyperreflective lesion in the outer retina, often associated with intraretinal fluid and, in some cases, SHRM, subretinal or sub-RPE fluid [43]. This hyperreflective lesion extends toward the RPE and is usually accompanied by a downward displacement of the outer retinal layers with a funnel shape. It frequently reaches or overlies a drusenoid or serous PED [44].

**Fig. 3:**
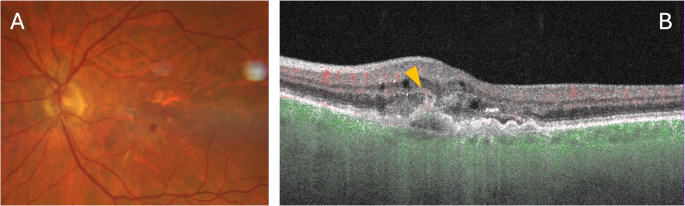
Multimodal imaging of the right eye from a patient with AMD and type 3 MNV. The color fundus photograph (**A**) reveals macular irregularities with pigmentary changes and a round-shaped hemorrhage. The OCTA B-scan (**B**) shows a hyperreflective lesion in the outer retina (orange arrowhead), accompanied by intraretinal and subretinal fluid. Flow within the lesion is confirmed by the superimposed red signal, consistent with type 3 macular neovascularization

In this context, OCTA can be a valuable tool to assess whether a hyperreflective lesion in the outer retina contains flow suggestive of type 3 MNV (Fig. 3). To confirm the presence of type 3 MNV, a 3 × 3 mm or 6 × 6 mm OCTA scan is recommended, as both provide sufficient macular coverage with high-resolution detail. The key step is evaluating cross-sectional OCTA images to detect flow within the hyperreflective lesion located in the outer retina. It is crucial, however, to rule out projection artifacts, as the hyperreflective lesion may falsely reflect flow from the SCP or DVC. In such cases, verifying the presence of flow at the same location in different layers can help identify and exclude projection artifacts. En face visualization is generally less informative for type 3 MNV, as it typically appears as a small, tuft-like capillary network in the ORCC slab, which does not exhibit the characteristic morphology of neovascular networks. Type 3 MNV should be differentiated from lesions such as deep retinal age-related vascular anomalies (DRAMA), which can present similar characteristics (please see below for details) [44].

OCTA can also be used to obtain quantitative parameters of MNV, including perfusion density, vessel length density, and neovessel diameter. Although quantitative variables may be informative, their clinical use remains controversial due to heterogeneity in methodologies and metrics. This review was not designed to address MNV quantification, as its primary aim was to provide practical guidelines for clinical practice.

### Geographic atrophy

While the management of GA is highly dependent on structural OCT including novel methods for image analyses [45, 46], OCTA may play a role in the evaluation of patients with GA. It has been extensively used to identify biomarkers linked to GA progression over time, with CC perfusion in the area surrounding GA recognized as a key marker—lower perfusion being associated with faster disease progression [47]. However, this application currently has limited clinical relevance. In practice, OCTA is primarily used to detect the presence of MNV that may complicate cases of GA.

MNV complicating GA may present with or without OCT signs of exudation, such as SRF, IRF, or SHRM. Non-exudative MNV associated with GA is typically type 1 and may be suspected when a shallow, irregular PED with a DLS is observed in a region without atrophy. To confirm the presence of type 1 MNV, a 3 × 3 mm or 6 × 6 mm OCTA scan is recommended, as both offer sufficient macular coverage and high-resolution detail. The diagnostic process begins with cross-sectional OCTA to detect flow between the RPE and Bruch’s membrane, corresponding to the hyperreflective material forming the DLS. En face OCTA then provides a comprehensive view of the lesion. However, in the presence of RPE atrophy, segmentation boundaries may be significantly distorted, and the RPE–RPEfit slab may not be reliably generated. In such cases, the ORCC slab can be a reasonable alternative, though it may also be affected by neuroretinal alterations due to GA. Another option is to use the CC slab, which is based on Bruch’s membrane as a reference—an anatomical landmark typically preserved even in the presence of RPE atrophy. The CC slab can be manually adjusted in thickness and position to encompass the PED and potentially enhance visualization of the underlying MNV. It is important to note that OCTA plays a key role in identifying MNV; however, its ability to determine the presence of exudation is limited, as structural OCT remains essential for this assessment.

GA may be complicated by exudative MNV as well. In such cases, the neovascularization is typically a type 2 MNV, extending above the RPE at the margins of the lesion. This should be suspected when SHRM is seen over areas of preserved RPE. In addition to *en face* visualization, detecting flow within the SHRM on OCTA B-scans is a key indicator of neovascular activity. In this scenario, identifying an exudative MNV is of major clinical importance, as it suggests the initiation of intravitreal anti-VEGF therapy, which is usually highly effective in these cases [48]. As previously discussed, the reliability of standard OCTA slabs may be affected by structural retinal changes associated with GA. Therefore, the use of the ORCC slab, or a manually adjusted CC slab—modified in both thickness and position—can enhance lesion visualization in this context.

## Non-exudative MNV in early/intermediate AMD

Neovascularization can complicate AMD even in the absence of OCT signs of exudation (such as SRF, IRF, serous PED or SHRM). Although treatment is typically not indicated in these cases, the follow-up schedule may be adjusted when such findings are identified—unlike in patients with early or intermediate AMD without evidence of non-exudative MNV.

Non-exudative MNV primarily includes two subtypes: type 1 and type 3 MNV.

Type 1 non-exudative MNV was initially characterized on FA as a late speckled hyperfluorescent lesion without well-defined borders, or as a hyperfluorescent plaque visible during the mid- and late-phases of ICGA, in the absence of fluid on structural OCT [49]. While FA and ICGA may detect type 1 non-exudative MNV, OCTA may be the best tool for this assessment because of its high sensitivity of detection and ease of use [50–52]. Studies have shown that OCTA can detect non-exudative MNV in up to 30% of patients with intermediate AMD. This finding aligns with earlier ICGA research indicating that approximately 25% of eyes with drusen show evidence of asymptomatic MNV [53, 54].

In a study following eyes with non-exudative MNV using structural OCT and OCTA over one year, the incidence of exudation was 21.1% in eyes with type 1 non-exudative MNV, compared to just 3.6% in those without [51] A subsequent study examining the 24-month risk of exudation found that eyes with subclinical MNV were 13.6 times more likely to develop exudation than those without [55] These findings underscore that eyes with documented non-exudative MNV have a significantly higher risk of progressing to exudative disease. OCTA, therefore, plays a valuable role in monitoring patients with intermediate AMD and can help guide follow-up scheduling.

Similar to its exudative counterpart, non-exudative type 1 MNV is usually suspected based on structural OCT findings. It appears as a shallow, irregular PED [30] containing a thick DLS, but without signs of SRF, IRF, or SHRM. To confirm the presence of type 1 MNV, a 3 × 3 mm or 6 × 6 mm OCTA scan is recommended, as both provide adequate macular coverage and high-resolution detail. Detection begins with cross-sectional OCTA to identify flow between the RPE and Bruch’s membrane, corresponding to the hyperreflective material forming the DLS. En face visualization then offers the best overview of the lesion. Two *en face* slabs may be used: the RPE-RPE fit slab and the ORCC slab. In the absence of neovessel components above the RPE—unlike in exudative type 1 MNV—the RPE–RPEfit slab may be preferred due to its superior contrast between the MNV and surrounding tissue.

Type 3 MNV can also be diagnosed in the absence of clear OCT signs of exudation (i.e., this entity referred as non-exudative type 3 MNV or nascent type 3 MNV) [56, 57], particularly IRF, which typically appears early in this context (i.e., stage 1 type 3 MNV). Suspicion arises when structural OCT reveals hyperreflective material in the outer retina without associated IRF. In these cases, a slight elevation or “pumping” of the neuroretina may be observed near the hyperreflective foci, suggesting the presence of minimal exudation—insufficient, however, to produce hyporeflective spaces indicative of IRF. In this scenario, OCTA becomes a valuable diagnostic tool. It can help determine whether a hyperreflective lesion in the outer retina contains flow suggestive of type 3 MNV, using either 3 × 3 mm or 6 × 6 mm scan areas. The critical step involves analyzing cross-sectional OCTA images to identify flow within the hyperreflective outer retinal lesion. As with exudative type 3 MNV, it’s essential to exclude projection artifacts, since these lesions can falsely appear to exhibit flow due to signals projected from the superficial or deep vascular complexes (SCP or DVC).

In these cases, anti-VEGF treatment is not typically indicated; however, a shorter follow-up interval may be advisable to allow early detection of exudation and timely initiation of treatment. It is important to emphasize that, although OCTA plays a role in identifying MNV, the evaluation of exudation relies primarily on structural OCT.

## Acquired vitelliform material in early/intermediate AMD

Vitelliform material can accumulate in the context of inherited retinal diseases—most notably in Best macular dystrophy [58]—but it is also observed in various acquired retinal conditions, including age-related macular degeneration (AMD), tractional maculopathies, and central serous chorioretinopathy.[59] In such acquired cases, these deposits are referred to as pseudovitelliform lesions or acquired vitelliform lesions (AVL) [59].

**Fig. 4:**
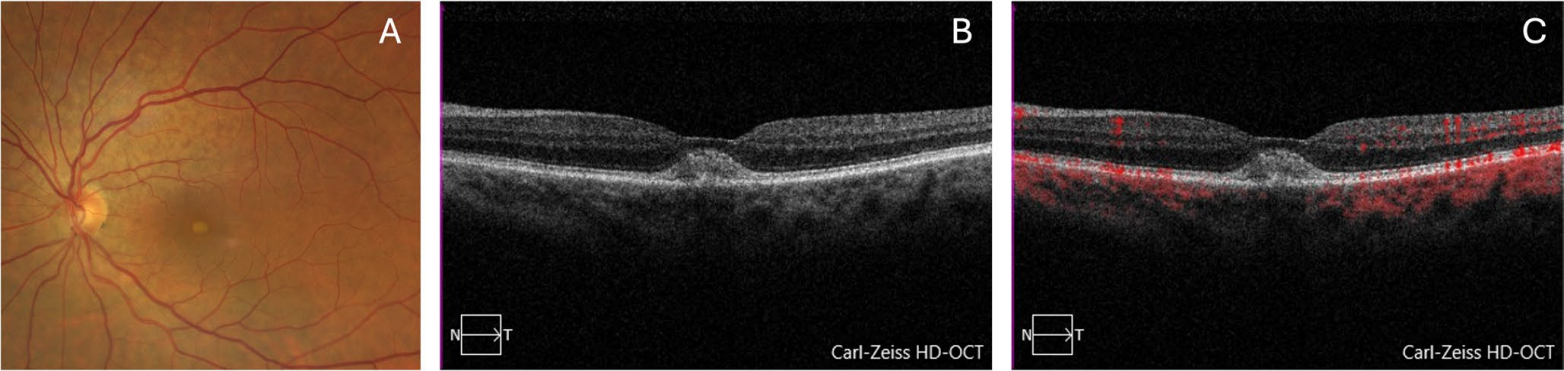
Multimodal imaging of the left eye in a patient with AMD and acquired vitelliform material. The color fundus photograph (**A**) shows reticular pseudodrusen predominantly in the superior macula and a hypopigmented subretinal lesion in the foveal region. On structural OCT (**B**), the foveal lesion appears as a hyperreflective material. The OCTA B-scan (C) demonstrates no detectable flow within the lesion, ruling out neovascularization and supporting the diagnosis of acquired vitelliform material associated with AMD

AVL appear as yellowish, slightly elevated changes on fundus examination, typically located beneath the fovea (Fig. 4). On structural OCT, they are characterized by the accumulation of hyperreflective material in the subretinal space (dome-shaped SHRM), often accompanied by an irregular RPE [60]. AVL are relatively common in patients with early or intermediate AMD and are typically associated with the presence of reticular pseudodrusen (RPD) [61, 62]. Eyes with intermediate AMD and AVL often demonstrate a higher prevalence of pachyvessels and increased choroidal thickness compared to those without AVL [62]. Interestingly, AVL may increase in size over time and subsequently undergo resorption. The coexistence of RPD and greater AVL height has been shown to significantly elevate the risk of atrophy developing at the lesion site following resorption [61].

OCTA plays a crucial role in this context for two main reasons: (i) AVL can be complicated by neovascularization, with neovessels infiltrating the pseudovitelliform material in approximately 12% of cases [60]; (ii) what appears to be SHRM resembling pseudovitelliform deposits may actually be associated with neovessels. Specifically, SHRM may result from type 1 MNV, in which case a flat, irregular PED is typically observed beneath the SHRM, or from type 2 MNV, where the SHRM itself contains neovascular elements.

To confirm the presence of neovascularization, a 3 × 3 mm or 6 × 6 mm OCTA scan is recommended. Evaluation should begin with cross-sectional OCTA to detect flow either within the SHRM or in a potential flat irregular PED beneath it. En face OCTA then provides the most comprehensive view of the lesion. The ORCC slab is often preferred, as it can effectively capture both the SHRM and any underlying flat PED.

It is important to emphasize that fundus autofluorescence (FAF) can aid in distinguishing AVL from exudative SHRM. AVL typically appears hyperautofluorescent, whereas exudative SHRM is usually iso- or hypoautofluorescent [63].

## Exudative intraretinal fluid in early/intermediate AMD or GA with no MNV

Exudative fluid has traditionally been associated with a neovascular origin. However, entities of non-neovascular exudative IRF have been recently observed and described.

In the absence of MNV, non-neovascular exudation is defined by the presence of newly detected intraretinal cystic spaces on structural OCT, accompanied by increased retinal thickness and displacement of adjacent retinal structures[64]. In these cases, anti-VEGF treatment may be avoided; however, the presence of exudative signs in the foveal region may justify treatment, which can be successful in selected cases [65].

**Fig. 5:**
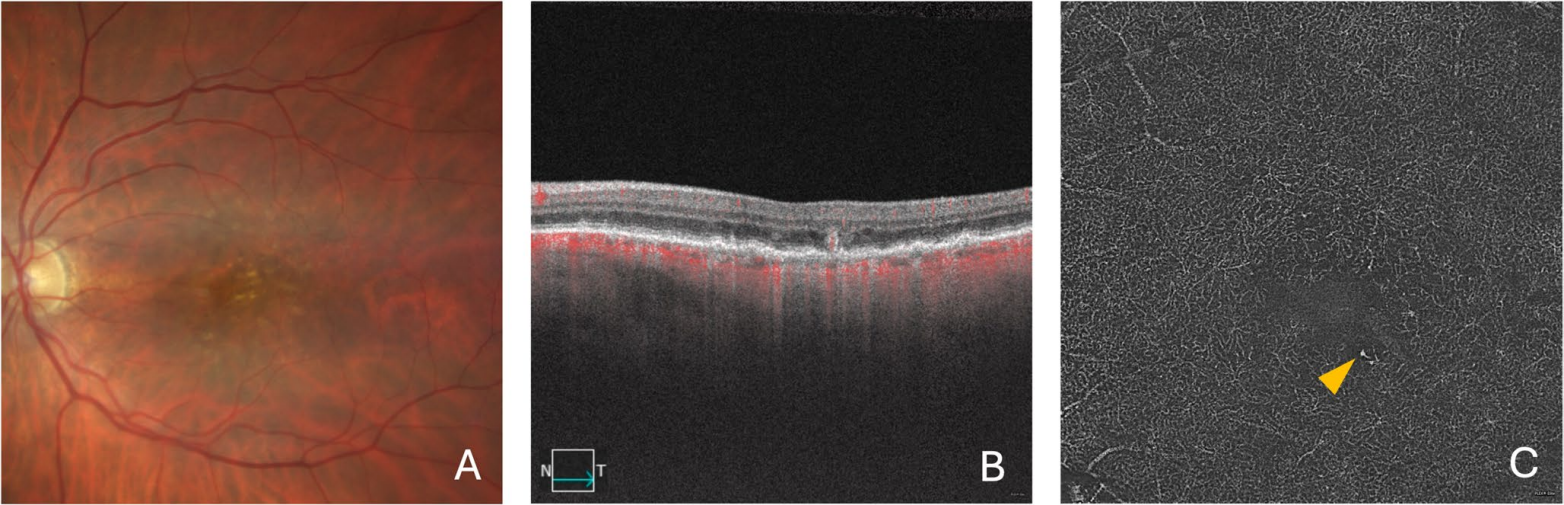
Multimodal imaging of the left eye in a patient with AMD and DRAMA. The color fundus photograph (**A**) reveals pigmentary alterations and drusen in the macular region. The OCTA B-scan (**B**) shows a DRAMA, characterized by a hyperreflective aneurysmal lesion with intralesional flow, associated with intraretinal fluid. The OCTA en face image segmented at the outer retina level (C) displays a well-defined, hyperreflective round lesion corresponding to the DRAMA (orange arrowhead)

Non-neovascular exudation tends to occur in an older population exhibiting classic features of AMD. In the latter setting, a chronic ischemic state in the retina may develop in areas with significant disruption of the Bruch’s membrane–RPE complex, likely due to functional insufficiency of the choriocapillaris [64, 66]. This insufficiency can elevate intraretinal VEGF levels that may eventually promote leakage from the DVC [64, 66]. This type of exudation may occur with or without associated microvascular anomalies, such as the recently described “deep retinal age-related microvascular anomalies” (DRAMA) [64, 67]. DRAMAs typically present as small-diameter perifoveal capillary dilations with hyperreflective walls located in the inner nuclear layer, or as multiple vascular outpouchings extending posteriorly into the Henle fiber layer, with reflectivity similar to surrounding normal retinal capillaries (Fig. 5) [67].

When exudative intraretinal fluid is suspected in non-neovascular AMD (i.e., early/intermediate AMD or GA), OCTA is essential for excluding the presence of MNV. In particular, type 3 MNV should be carefully ruled out, as it may initially present with IRF. Additionally, the presence of DRAMAs should be eventually confirmed, especially in the presence of oval-shaped hyperreflective lesions on structural OCT. OCTA is typically able to detect flow within these lesions, supporting the diagnosis. Accurate diagnosis is crucial, as DRAMAs have been found to respond to anti-VEGF therapy [68, 69].

While the presence of IRF in this context is generally attributed to exudation, it is important to acknowledge that a portion of this fluid may also originate from transudation. This concept is well established for subretinal fluid in eyes with intermediate AMD or GA (i.e., see paragraph below), and it may similarly apply to IRF in certain cases.

## Non-exudative subretinal fluid in intermediate AMD or GA

**Fig. 6:**
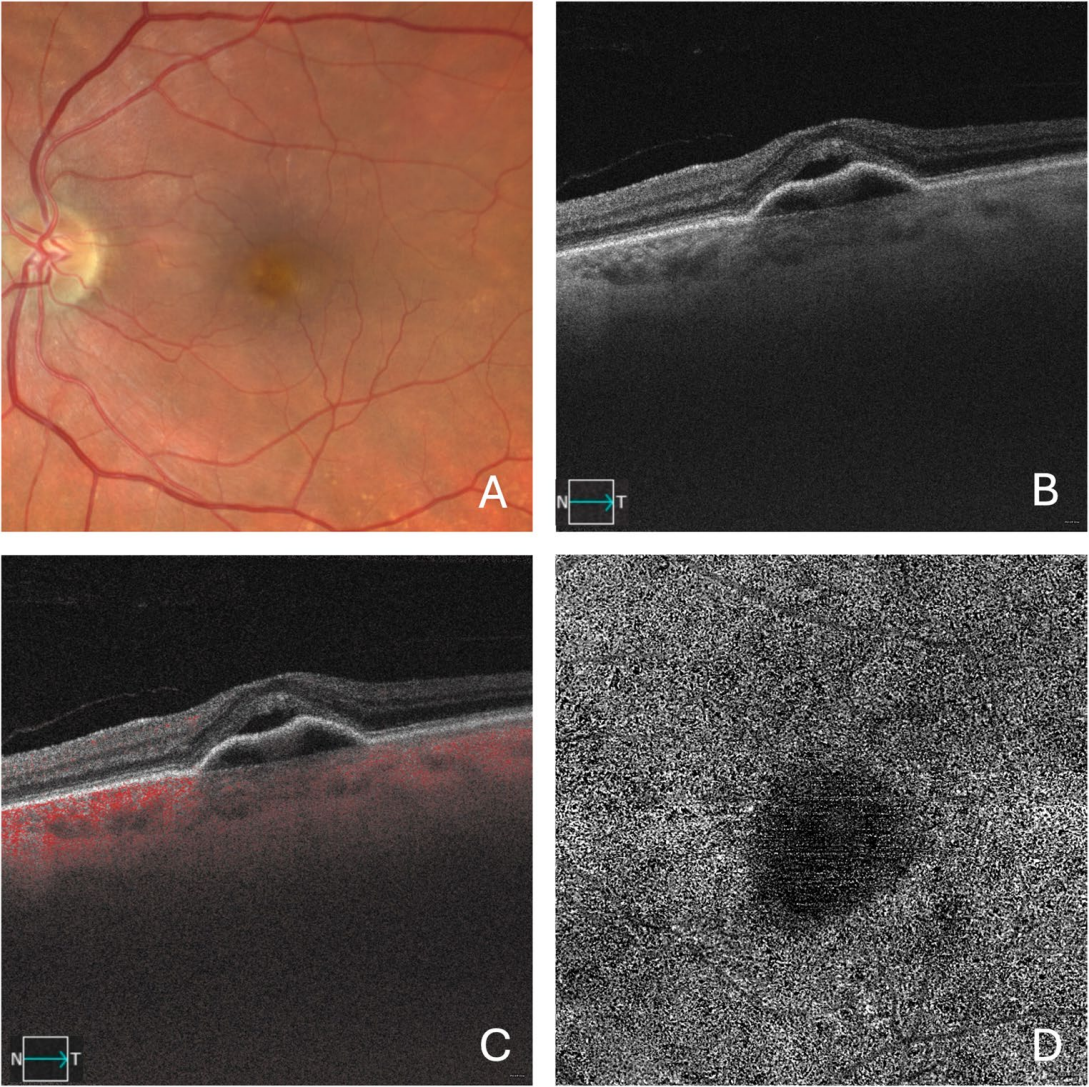
Multimodal imaging of the left eye in a patient with AMD and non-exudative subretinal fluid. The color fundus photograph (**A**) reveals pigmentary alterations and drusen in the macular region. The OCTA B-scan (**B**) shows a large pigment epithelium detachment, associated with subretinal fluid. The OCTA B scan image (**C**) does not show any evidence of flow within this RPE detachment. Accordingly, the en face image segmented at the ORCC level (C) displays an absence of evident neovascularization

Non-exudative SRF was first described by Hilely and colleagues [70]. This finding is not uncommon and is typically associated with large drusenoid PEDs, where the SRF is usually localized at the apex or along the edge of the PED (Fig. 6). It has been proposed that the presence of SRF may be related to increased separation between the RPE and the CC, resulting in RPE hypoperfusion and subsequent dysfunction [70].

In this context, it is crucial to differentiate between a large drusenoid PED and a serous PED. Structural OCT can aid in this distinction, as drusenoid PEDs typically exhibit higher internal reflectivity, whereas serous PEDs tend to appear hyporeflective. However, OCTA plays a key role in further characterizing these lesions. In cases of serous PED, MNV may be present beneath the PED or, more commonly, at its edge. Therefore, it is important to carefully examine for the presence of a shallow, irregular PED at the margin of the suspected serous PED and assess for any flow signal on OCTA that may indicate underlying type 1 MNV. However, in cases of large serous PED, OCTA segmentation can sometimes be inaccurate; in such situations, FA and ICGA remain essential tools for the detection of MNV.

In the presence of non-exudative subretinal fluid in intermediate AMD or GA, intravitreal anti-VEGF treatment is typically not required.

### Non-exudative intraretinal fluid in early/intermediate AMD or GA

Non-exudative IRF may be associated with degenerative intraretinal “pseudocysts” or “cavitations” that typically occur in areas of outer retinal atrophy, with or without accompanying RPE atrophy [71, 72]. Structural OCT can aid in distinguishing these lesions from exudative fluid.[73] Degenerative pseudocysts tend to exhibit significantly lower circularity, lack a perfusion signal in the peri-cystic space, and less frequently display peri-cystic hyperreflective foci [73]. Nevertheless, OCTA remains essential for ruling out exudation originating from MNV or retinal microvascular anomalies (i.e., as discussed in the sections above). In the presence of non-exudative intraretinal fluid in intermediate AMD or GA, intravitreal anti-VEGF treatment is typically not required.

## Conclusions

OCTA has revolutionized the imaging landscape in AMD, offering non-invasive, high-resolution, and depth-resolved visualization of retinal and choroidal vasculature. Its integration into routine clinical practice enables more precise diagnosis, differentiation, and monitoring of both neovascular and non-neovascular manifestations of AMD across a broad spectrum of disease stages. This review provides a pragmatic framework to guide clinicians in selecting the most appropriate OCTA strategies for different clinical scenarios, including neovascular AMD subtypes, GA, pseudovitelliform lesions, and exudative changes without evident neovascularization.

Given the heterogeneity of AMD and the complexity of its presentations, personalized imaging protocols tailored to specific anatomical and pathological contexts are essential. Advances in image quality and the adoption of wider field-of-view scan patterns may enable the assessment of retinal and choroidal vasculature beyond the central macula, this allowing for the detection of non-macular lesions, including MNVs located in the peripapillary region, which are not uncommon in AMD. Accurate interpretation of OCTA findings, combined with structural OCT, enhances clinical decision-making and may ultimately improve patient outcomes. As technology continues to evolve and evidence grows, OCTA is poised to play an increasingly central role in the comprehensive management of AMD. In this setting, the increasing integration of artificial intelligence into OCTA devices may further enhance the clinical use of this technology by enabling the selection of the most appropriate segmentation strategy for each case. In addition, an increasing number of longitudinal OCTA studies in AMD will help define the optimal management strategy for individual cases and may identify biomarkers of disease progression.

## Appendix

European Young Retinal Imaging and retina Study Group:

- Daniela Bacherini, Department of Neurosciences, Psychology, Drug Research, and Child Health, Eye Clinic, University of Florence, Azienda Ospedaliero Universitaria Careggi, Florence, Italy.
- Enrico Borrelli, Department of Surgical Sciences, University of Turin, Turin, Italy.
- Rosa Dolz-Marco, Unit of Macula, Oftalvist Clinic, 46004 Valencia, Spain.
- Roberto Gallego-Pinazo, Unit of Macula, Oftalvist Clinic, 46004 Valencia, Spain.
- Rodolfo Mastropasqua, Department of Neurosciences, Imaging and Clinical Sciences, University “G. d’Annunzio” Chieti-Pescara, Chieti, Italy.
- Gregor S. Reiter, Department of Ophthalmology and Optometry, Medical University of Vienna, Vienna, Austria.
